# Cerebello-Cerebral Pathways Contribute to Written Word Production

**DOI:** 10.1162/nol.a.10

**Published:** 2025-08-14

**Authors:** Romi Sagi, Sivan Jossinger, J. S. H. Taylor, Kyriaki Neophytou, Brenda Rapp, Kathleen Rastle, Michal Ben-Shachar

**Affiliations:** The Gonda Multidisciplinary Brain Research Center, Bar-Ilan University, Ramat-Gan, Israel; Division of Psychology and Language Sciences, University College London, London, UK; Department of Cognitive Science, Johns Hopkins University, Baltimore, MD, USA; Department of Psychology, Royal Holloway, University of London, London, UK; Department of Neurology, Johns Hopkins Medicine, Baltimore, MD, USA

**Keywords:** cerebellum, diffusion MRI, spelling, superior cerebellar peduncle, white matter, written word production

## Abstract

Written language production is a fundamental aspect of daily communication, yet the neural pathways supporting it are far less studied than those for spoken language production. This study evaluated the contributions of speech-production pathways to written word production, specifically focusing on the central processes of word spelling rather than the motor production processes that support handwriting. Seventy-three English-speaking, neurotypical adults completed a spelling-to-dictation task and underwent diffusion MRI scans. The bilateral cerebello-thalamo-cortical pathways (CTC) and frontal aslant tract (FAT) were identified in individual participants using probabilistic tractography and automated segmentation tools. Fractional anisotropy (FA) values were computed along the trajectory of each tract and entered into correlation analyses with the spelling accuracy scores. A significant correlation was found between spelling accuracy scores and FA in the left CTC, which connects the left cerebellar hemisphere with the right cerebral hemisphere. This effect remained significant after controlling for spoken production measures. A similar trend was observed in the right homologous tract. In contrast, no significant correlations were identified between spelling accuracy scores and FA in the bilateral FAT. These findings demonstrate, for the first time, the involvement of cerebello-cerebral connections in spelling processes, aligning with the growing recognition regarding the role of the cerebellum in higher-order language functions. This effect did not generalize to the FAT, which may be relevant for more peripheral aspects of language production.

## INTRODUCTION

Producing written language is a highly complex human skill, involving a distributed network of cortical, subcortical, and cerebellar brain regions (Planton et al., 2013; Purcell, Turkeltaub, et al., 2011). Although written language production is essential for everyday communication and functioning, the neural pathways supporting it have been far less studied compared to those for speech production. Studies of spoken language have demonstrated the involvement of an extensive network of white matter pathways in mediating fluent and efficient speech production, including the arcuate fasciculus, frontal aslant tract (FAT), and cerebellar pathways (Ackermann et al., 2007; Connally et al., 2014; Dragoy et al., 2020; Hickok, 2012; Jossinger et al., 2024; Kronfeld-Duenias et al., 2016; Saur et al., 2008; Simonyan & Fuertinger, 2015). These white matter pathways are also known to connect brain regions associated with written language production, as identified in functional magnetic resonance imaging (fMRI) and lesion studies (Baldo et al., 2018; Planton et al., 2013; Purcell, Turkeltaub, et al., 2011; van Dun et al., 2016). However, the direct involvement of some of these white matter structures, specifically the cerebello-cerebral pathways and FAT, has yet to be studied in the context of written word production. To address this gap, we assessed the associations of these pathways with spelling performance in healthy adult English speakers.

The production of written words relies on the integration of multiple neurocognitive processes, which can be roughly divided into *central* and *peripheral* components (Tao & Rapp, 2025). Central processes are not dependent on the output mode (e.g., typing, handwriting, or oral spelling). They include mental retrieval of an abstract lexical orthographic representation or its construction through the mapping of phonemes to graphemes (Tainturier & Rapp, 2001), as well as temporary maintenance of information about grapheme identities and their order in an *orthographic working memory* system (Caramazza et al., 1987). Peripheral processes depend on the output mode and include programming appropriate motor actions for executing typed or handwritten letters, or retrieving letter names for oral spelling. As we further explain below, the distinction between central and peripheral processes is particularly relevant to the discussion regarding the involvement of cerebellar pathways in written word production (van Dun et al., 2016).

The importance of the cerebellum in written word production is strongly supported by fMRI data. The cerebellum is one of the most consistently activated structures during written word production tasks, as demonstrated in activation likelihood estimation (ALE) meta-analyses (Purcell, Turkeltaub, et al., 2011; Planton et al., 2013). It was initially assumed that the cerebellum is predominantly involved in peripheral writing processes (Dow & Moruzzi, 1958), a view supported by disrupted graphomotor processing following cerebellar lesions (De Smet et al., 2011; Mariën et al., 2007; Silveri et al., 1997, 1999). However, other cerebellar lesion studies described impairments to central aspects of spelling, such as lexical, sublexical, and orthographic working memory processes (Fabbro et al., 2000, 2004; Mariën et al., 1996, 2009; Paul et al., 2022). These findings align with the growing recognition that the cerebellum plays an important role in higher-order language functions, such as phonological or semantic processing (De Smet et al., 2007; King et al., 2019; LeBel & D’Mello, 2023; Schmahmann, 2019; Stoodley et al., 2012; Vias & Dick, 2017). Here, we assess the contribution of the cerebello-cerebral pathways to *central* aspects of written word production, focusing on spelling as a composite skill that probes multiple central written word production processes (Purcell, Turkeltaub, et al., 2011).

A complementary approach to the functional imaging and lesion studies reviewed above uses diffusion MRI (dMRI) to study white matter associations with spelling. Spelling requires the transfer of information within distributed brain regions and thus relies heavily on the white matter pathways connecting them. So far, only a limited number of dMRI studies have assessed the associations between white matter pathways and spelling processes, primarily focusing on language pathways within the classic model of language connectivity (Dick et al., 2014). These studies have shown that both ventral and dorsal streams are involved in spelling: ventrally, associations with spelling were detected in the left inferior longitudinal fasciculus and uncinate fasciculus, and dorsally, in left arcuate fasciculus and the right superior longitudinal fasciculus (Banfi et al., 2019; Cheema et al., 2023; Gebauer et al., 2012; Sagi et al., 2024). However, other pathways that feature in the extended network for language production (Dick et al., 2014), including cerebellar tracts and the FAT, have not yet been investigated in dMRI studies of written word production.

In terms of cerebellar connectivity, we focus our analysis on the principal output pathway from the cerebellum to the cerebrum, the cerebello-thalamo-cortical (CTC) tract. This tract originates in the dentate nucleus of each cerebellar hemisphere, decussates (crosses over) at the midbrain level, and synapses in the contralateral thalamus. From there, thalamocortical projections continue to the contralateral cerebral cortex (Palesi et al., 2015). The potential involvement of the CTC in written word production is supported by these thalamocortical projections. Cortically, the CTC has been shown to project to motor, prefrontal, parietal, and temporal association areas (Behrens et al., 2003; Clower et al., 2001; Kelly & Strick, 2003; Middleton & Strick, 2000; Palesi et al., 2015; Stepniewska et al., 1994). These projections are highly consistent with the cortical network associated with written word production, which includes the inferior frontal gyrus (IFG), the supplementary motor area (SMA), the superior parietal lobule, the ventral occipitotemporal cortex, and the superior temporal gyrus (Planton et al., 2013; Purcell, Turkeltaub, et al., 2011). Subcortically, the CTC synapses in the ventral lateral nucleus of the thalamus, which is consistently active in fMRI studies of written word production (Planton et al., 2013; Purcell, Turkeltaub, et al., 2011). Prior dMRI studies have demonstrated the contribution of the CTC to several aspects of *spoken* language production, showing associations between its microstructural properties and reading aloud in children and adolescents (Bruckert et al., 2020; Travis et al., 2015), naming in post-stroke aphasia (Keser et al., 2023), and phonemic fluency in healthy adults (Jossinger et al., 2024). Whether this tract is involved in *written* word production remains to be examined.

Along with the cerebello-cerebral pathways, the FAT may also play a role in producing written words, although it is less likely to be involved in its central aspects, as we explain below. The FAT connects the IFG with the SMA and pre-SMA, regions within the established written word production network (Planton et al., 2013; Purcell, Turkeltaub, et al., 2011). This pathway has been extensively implicated in speech production studies (Dick et al., 2019); for instance, electrical stimulation of the left FAT during awake surgery in patients with glioma has been shown to induce intraoperative stuttering (Kemerdere et al., 2016), inability to complete a sentence (Dragoy et al., 2020), or speech arrest (Kinoshita et al., 2015). dMRI studies have demonstrated differences in the microstructural properties of the bilateral FAT between adults who stutter and control participants (Kronfeld-Duenias et al., 2016), and they have shown associations of the bilateral FAT with speaking rate in healthy adults (Jossinger et al., 2024). Converging evidence, reviewed by Dick et al. (2019), suggests a domain-general role for the FAT in planning, timing, coordination, and competition mechanisms of voluntary motor movement sequences, which are essential for writing, but less relevant to central spelling processes. In sum, we expect that the FAT may be involved in peripheral (but not necessarily in central) aspects of written word production.

Here, we investigated the associations between spelling performance and the microstructural properties of the CTC and FAT. Healthy English-speaking adults completed a difficult spelling-to-dictation task designed to assess their accuracy in written spelling. The task was untimed, and participants were allowed to correct their responses to minimize peripheral errors. To obtain a broad range of spelling accuracy scores and avoid a ceiling effect, the task featured words with complex, one-to-many phoneme-to-grapheme mappings. All participants underwent dMRI scanning, in addition to the behavioral tasks. We used automated segmentation tools to reconstruct the bilateral CTC and FAT in individual participants, using constrained spherical deconvolution (CSD) modeling of the diffusion data coupled with probabilistic tractography. This approach allowed us to follow the CTC fibers through their decussation to the contralateral hemisphere (Jossinger et al., 2024). Subsequently, neurocognitive associations were assessed between spelling performance and microstructural measures of the tracts. To assess the potential contribution of spoken production to the white matter associations with spelling, we included Rapid Automatized Naming (RAN; Wagner et al., 2013) in the analyses.

We hypothesized that the CTC would show correlations with spelling accuracy scores, given the documented role of the cerebellum in central aspects of written language production (e.g., Fabbro et al., 2000, 2004; King et al., 2019; Mariën et al., 1996, 2009; Nicolson & Fawcett, 2011; Paul et al., 2022; Schmahmann, 2019; Stoodley et al., 2012; Vias & Dick, 2017). We anticipated stronger spelling associations with the right CTC (connecting the right cerebellar hemisphere with the left cerebral cortex), given that the right cerebellar hemisphere is more consistently linked to writing and language functions across studies and methodologies (e.g., lesion studies: De Smet et al., 2011; Mariën et al., 2007, 2009; Paul et al., 2022; Silveri et al., 1999; fMRI studies: Planton et al., 2013; Purcell, Turkeltaub, et al., 2011), and because the cortical network associated with spelling is left-dominant (Planton et al., 2013; Purcell, Turkeltaub, et al., 2011; Tao & Rapp, 2025). Finally, we hypothesized that the association between tract properties and spelling would remain significant when we control for spoken production. In contrast, since the FAT is primarily associated with peripheral aspects of speech production (Dick et al., 2019), we hypothesized that it is less directly involved in central spelling processes. Therefore, we did not expect anisotropy in the FAT to show associations with spelling scores.

## MATERIALS AND METHODS

### Participants

Seventy-three neurotypical university students participated in this study (see Table 1). All participants were native English speakers, right-handed, and did not have a history of diagnosed learning disabilities or neurological conditions. They were recruited as part of a larger research project that included an extensive cognitive assessment and MRI scans and were paid for their participation. Written informed consent was obtained from all participants before the study. The study was approved by the Ethics Committee of Royal Holloway, University of London, where all data were collected. Some data from this sample and its subsamples were included in other independent analyses published elsewhere (Bathelt et al., 2024; Rastle et al., 2021; Sagi et al., 2024; Taylor et al., 2017, 2019; Yablonski et al., 2019). However, the analyses reported here are entirely new.

**Table 1.** Sample characteristics (*N* = 73)

	Mean	*SD*	Range
Demographics			
Age (years)	21	4.2	[19, 35]
Gender	57F/16M	–	–
Behavioral scores			
Spelling accuracy (ratio)	0.41	0.234	[0.075, 0.975]
RAN Letters (SS)	8.30	2.947	[1, 15]
RAN Digits (SS)	9.03	3.184	[1, 16]
RAN subtests averaged	8.66	2.966	[1, 15.5]

*Note*. RAN = rapid automatized naming, SS = age-scaled scores, *SD* = standard deviation, F/M = female/male.

### Behavioral Measures

#### Written word production: Spelling-to-dictation

**Figure 1.**
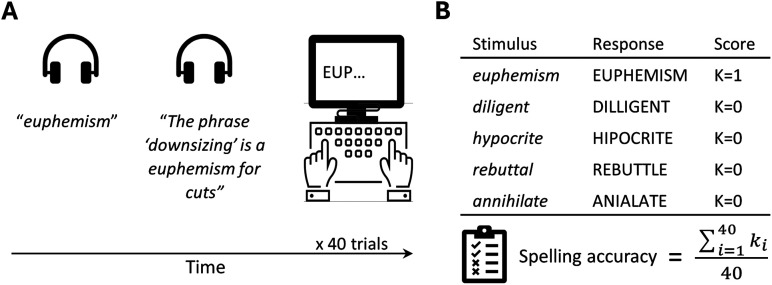
The spelling-to-dictation task. (A) Behavioral paradigm: Participants typed each word after listening to it in isolation and in the context of a carrier sentence. The stimuli included 40 low-frequency English words, each 8–10 letters long, with one-to-many phoneme-to-grapheme mappings (adapted from Burt & Tate, 2002). (B) Scoring example: Each response was classified as either correct or incorrect. Spelling accuracy was calculated for each participant as the proportion of correct items out of the total number of trials.

Participants underwent a typed spelling-to-dictation task to assess their written spelling performance (see also Sagi et al., 2024; Ulicheva, Coltheart, et al., 2021; Ulicheva, Marelli, & Rastle, 2021). The task consisted of 40 trials in which participants heard an English word via headphones, first in isolation and then as part of a contextual carrier sentence (e.g., “Euphemism—The phrase ‘downsizing’ is a euphemism for cuts”; see Figure 1A). Participants were instructed to type the word on a standard QWERTY keyboard. The typed response appeared on the screen and participants could use backspace to correct it. There was no time limit per trial. When they were ready, participants pressed the Enter button to move on to the next trial. Stimuli were adapted from Burt and Tate (2002) and included long (8–10 letters), low-frequency words with one-to-many phoneme-to-grapheme mappings to avoid a ceiling effect in the spelling accuracy of this group of neurotypical university students. The auditory stimuli were recorded by a female native speaker of Southern British English. Spelling accuracy scores were calculated for each participant as the proportion of correct items out of the total number of trials. To this end, responses were considered correct only if they were completely accurate (Figure 1B). A fine-grained analysis of spelling errors in this dataset was reported in a previous study (Sagi et al., 2024).

#### Spoken production: RAN

Participants completed two RAN subtests: Digits and Letters from the Comprehensive Test of Phonological Proceessing—Second Edition (CTOPP-2; Wagner et al., 2013). In each subtest, participants were instructed to name aloud a matrix of digits or letters as quickly and accurately as possible, and the time they took to complete each matrix was measured. Times were scaled according to the norms of the age group (mean = 10, *SD* = 3), yielding a score between 1 (poor performance) and 17 (excellent performance). Given that the digit and letter scores were highly correlated (Pearson’s *r* = 0.87), we averaged the letter and digit scaled scores for each participant to get a more robust measure of speeded spoken production. This averaged scaled score was used in further analyses (see Table 1 for RAN subtest and averaged scaled scores).

### MRI Data Acquisition

MRI data were obtained using a 3T Siemens Trio scanner with a 32-channel head coil (Siemens Medical Systems, Erlangen, Germany). The MRI protocol comprised anatomical and diffusion imaging sequences, as described below. Functional MRI sequences were also included in the scan protocol as part of a larger study, but those are not included in the current analyses.

#### T1 image acquisition

High-resolution T1-weighted anatomical images were acquired through a magnetization-prepared rapid acquisition gradient echo (MPRAGE) sequence, with the following parameters: Repetition time (TR) = 2,250 ms, echo time (TE) = 2.99 ms, flip angle = 9°, slice thickness = 1 mm, voxel size = 1 × 1 × 1 mm.

#### Diffusion-weighted image acquisition

A standard dMRI protocol was applied using a single-shot spin-echo diffusion-weighted echo-planar imaging (DW-EPI) sequence. Imaging parameters included 63 axial slices, each 2 mm thick with no gap, with a field of view of 192 × 192 mm and a matrix size of 96 × 96, providing a cubic resolution of 2 × 2 × 2 mm. The acquisition included 64 diffusion-weighted volumes (b = 1,000 s/mm^2^) and one reference volume (b = 0 s/mm^2^) using a standard diffusion direction matrix. The entire dMRI sequence lasted 8 min 52 s.

### dMRI Data Analysis

All processing steps of the dMRI data were conducted in the native space of each participant. Initially, we preprocessed the raw diffusion data and fitted local CSD models to the raw data, at the voxel level. Subsequently, we executed whole-brain probabilistic tractography, based on the voxel-wise CSD models. Lastly, we segmented the desired white matter pathways with automated tools and quantified their microstructural properties. Each step is described in more detail below. These analyses were implemented in MATLAB 2012b (MathWorks, 2012).

#### dMRI preprocessing

The dMRI data was processed using the mrDiffusion open-source software (Dougherty et al., 2006). The preprocessing pipeline included the following stages, each performed within the individual native space: (1) rotating T1 images to the anterior commissure–posterior commissure (AC-PC) orientation using a rigid transformation; (2) correcting motion and eddy-current distortions in DW-EPI data (Rohde et al., 2004); (3) registering diffusion-weighted volumes to the non-diffusion-weighted (b0) volume; (4) aligning the b0 image with the T1 image using a rigid body mutual information maximization algorithm (implemented in SPM8; Wells et al., 1996); (5) applying the combined transform from motion correction, eddy-current correction, and anatomical alignment to the raw diffusion data, while maintaining the original voxel size (2 × 2 × 2 mm); and (6) adjusting the table of gradient directions accordingly, to fit the resampled diffusion data (Leemans & Jones, 2009).

#### dMRI model fitting

Diffusion data in each voxel was modeled twice: first with a tensor model, which was used to compute microstructural properties, and second with CSD, which was used as the basis for tractography. The tensor model was fitted to the data using mrDiffusion with a standard least-squares algorithm. This model was utilized to calculate fractional anisotropy (FA) at each voxel. FA was calculated as the normalized standard deviation of the three estimated eigenvalues of the principal diffusion coefficients extracted from the tensor model (Pierpaoli & Basser, 1996). This scalar, ranging from 0 to 1, quantifies the extent to which water diffusion is faster along one direction compared to the others (0 = fully isotropic, no directional preference; 1 = strong preference toward a single direction). The CSD model was computed using the MRtrix3 suite of tools (Tournier et al., 2019). This model estimates a fiber orientation distribution function (fODF) within each voxel, which was further used as the basis for probabilistic fiber tracking. Specifically, we used the dhollander algorithm, implemented within the dwi2response function, to estimate diffusion response functions (Dhollander et al., 2016; Dhollander & Connelly, 2016). Based on the response functions estimated within the white matter and cerebrospinal fluid, we estimated fODFs by applying CSD with up to eight spherical harmonics (lmax = 8; Tournier et al., 2004, 2007). This step was carried out using the msmt_csd algorithm (Jeurissen et al., 2014), implemented within the dwi2fod function.

#### Whole-brain tractography

Probabilistic whole-brain tractography was performed based on the voxelwise CSD models (described above), using the iFOD2 algorithm, implemented in the MRtrix3 function tckgen. A whole-brain white matter mask was created for each participant from their structural T1 image using the 5ttgen (utilizing FSL tools) for whole-brain segmentation. The tracking process was initiated from 500,000 random seeds within the white matter mask, applying the following constraints: fODF amplitude threshold of 0.1; maximum angle of 45° between successive steps; step size of 0.85 mm; streamline length between 50 mm to 200 mm, with truncation of streamlines extending beyond the white matter mask. The resulting whole-brain tractograms, each containing 500,000 streamlines, were further segmented to identify the tracts of interest, as described below.

#### Automatic tract segmentation and quantification

Tract identification and quantification of diffusion parameters were carried out by the Automatic Fiber Quantification package (AFQ; Yeatman et al., 2012). Tracts of interest were segmented individually, in the native space of each participant, using a multiple region of interest (ROI) approach. In this method, specific pathways are isolated by intersecting the whole-brain tractogram with individually placed ROIs. Specifically, ROIs were predefined once (on the Montreal Neurological Institute T2 template), then projected to the native space of each individual. The resulting individual ROIs were intersected with the individual whole-brain tractogram using logical operations.

To identify the bilateral CTC, we used the protocol described in Jossinger et al. (2024) which enables segmenting the CTCs including their decussation to the contralateral cerebral hemisphere (see Figure S1 in the Supporting Information, available at https://doi.org/10.1161/nol.a.10, for the anatomical trajectory of the CTCs and locations of ROIs). To identify the bilateral FAT, we used the protocol described in Kronfeld-Duenias et al. (2016). Both protocols are implemented in AFQ (and publicly available at: https://github.com/yeatmanlab/AFQ/tree/master/cp-prob_segmentation; https://github.com/yeatmanlab/AFQ/tree/master/aslant). For each tract, an automatic cleaning procedure was applied, excluding streamlines that were longer than 4 standard deviations from the mean fiber length or that spatially deviated more than 4 standard deviations from the core of the tract (i.e., from the mean *x*, *y*, *z* coordinates at each node along the tract; Yeatman et al., 2012).

Finally, FA profiles were delineated by extracting FA values at 30 equidistant nodes along each tract of interest for each participant. At each node, FA was determined by a weighted average, with streamlines closer to the core weighing more heavily than those further from the core. FA was quantified within the central portion of the tracts enclosed between the two ROIs, where trajectories of the tracts are more consistent across individuals (Yeatman et al., 2012).

#### Quality assurance and outlier detection

We first calculated tract-FA values, for each participant and each tract, defined as the mean FA across the 30 nodes along a particular tract. An individual tract was considered an outlier if the tract-FA value deviated by more than ±3 standard deviations from the mean tract-FA (averaged across individuals). We further inspected the tractograms in the native space of each participant, to determine if they matched the expected trajectory based on anatomical descriptions (Catani et al., 2012; Oishi et al., 2010) or based on previous findings using similar segmentation protocols in an independent sample (Jossinger et al., 2024; Yablonski et al., 2021). For visual inspection of the tracts, we used mrDiffusion and Quench, a 3D visualization tool (Akers, 2006).

### Brain-Behavior Correlation Analyses

The associations between spelling performance and FA were assessed by two-tailed Spearman’s rank-order correlation coefficients. Spearman’s correlations were used since the distribution of spelling accuracy scores across participants significantly deviated from normality, as indicated by a Shapiro-Wilk normality test (W = 0.94, *p* = 0.003). First, we calculated, for each tract, the correlation between spelling accuracy scores and tract-FA. We handled multiple comparisons across the four tracts by controlling the false discovery rate (FDR) at a level of 0.05 (Benjamini & Hochberg, 1995). Since diffusivity values have been shown to vary substantially along cerebral and cerebro-cerebellar tracts (Jossinger et al., 2024; Palesi et al., 2016; Yeatman et al., 2012), we then assessed the correlations between spelling accuracy scores and FA along the tract profile (for similar approaches, see Yablonski et al., 2021; Yeatman et al., 2012). Significance was corrected for 30 comparisons (30 nodes along the tract profile) using a nonparametric permutation method, yielding a family-wise error (FWE)-corrected alpha value of 0.05 (Nichols & Holmes, 2002). Accordingly, a significant correlation was determined only if there was a sufficiently large cluster of consecutive nodes, each showing a correlation with *p*_uncorrected_ < 0.05. The required minimal cluster size (number of nodes) was computed by a permutation algorithm (Nichols & Holmes, 2002). The same procedure was applied to calculate associations between FA and RAN scores. In this case, a two-tailed Pearson’s linear correlation was used, because the distribution of the RAN scores did not significantly deviate from normality (Shapiro-Wilk normality test: W = 0.97, *p* = 0.060).

### Specificity of Brain-Behavior Associations

#### Lateralization

To evaluate the lateralization of white matter associations with spelling, we conducted a linear mixed-effects (LME) analysis. Tract-FA values for each participant and each tract were entered as the dependent variable. The intercept per Participant was set as a random factor. As fixed factors, we had Hemisphere (left, right), Tract (CTC, FAT), and Spelling accuracy (accuracy scores), including all their interactions. For the purpose of assessing lateralization, the interaction effects of Spelling accuracy × Hemisphere and Spelling accuracy × Tract × Hemisphere were of particular interest. The LME model was calculated with the MATLAB 2022a function fitlme using effects coding, and model coefficients were tested for significance using Student’s *t* tests.

#### Written versus spoken production measures

To assess the contribution of spoken production to the white matter correlations detected with spelling scores, we conducted multiple regression analyses including both written and spoken production measures. Cluster-FA (i.e., mean FA across the nodes within the cluster) was set as the dependent variable, and Spelling accuracy and RAN scores were entered as the predictor variables. To further evaluate the unique contribution of each predictor, we conducted nested model comparisons, comparing the full model (Spelling + RAN) to reduced models containing a single predictor, either Spelling or RAN. These comparisons allowed us to test whether adding each predictor significantly improved model fit over the other. In addition, to control for multicollinearity in behavioral measures, we calculated the variance inflation factors (VIF) for each variable (Johnston et al., 2018). Regression models and VIF values were calculated using JASP (JASP Team, 2022).

## RESULTS

### Behavioral Results

Spelling accuracy and RAN scores were broadly distributed across participants, spanning the entire range (see Table 1 for descriptive statistics). The average time per trial in the spelling task was 7.4 s (*SD* = 3.6). A detailed analysis of spelling errors in this dataset, conducted in a previous study (Sagi et al., 2024), revealed that the errors were predominantly phonologically plausible (e.g., spelling “dissuade” as DISWAYED; see Figure 1B for more examples; and Sagi et al., 2024, for a full description of the error analysis). Spelling accuracy and RAN scores were not statistically correlated (Spearman’s *r* = −0.02; see Figure S2).

### Tract Identification

**Figure 2.**
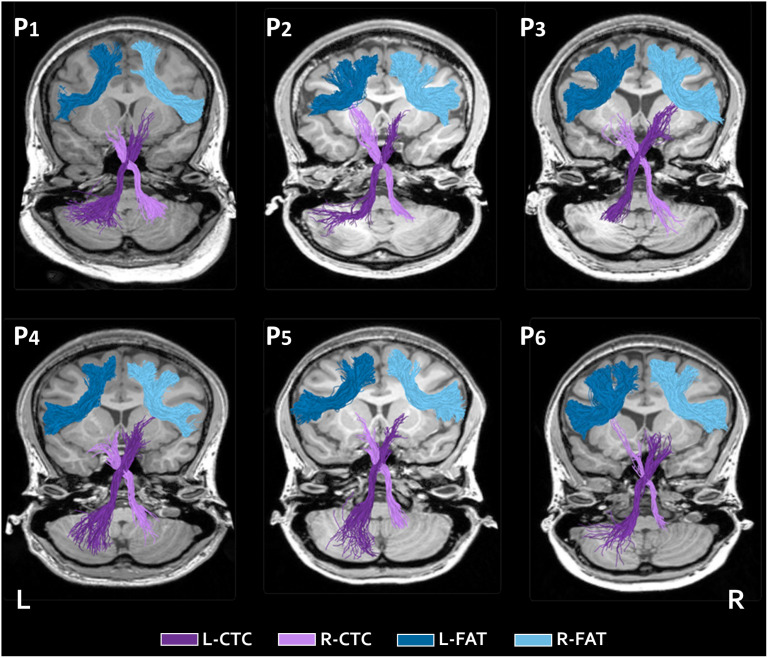
Tracts of interest. Bilateral tracts identified with probabilistic tractography are displayed in six representative participants, overlaid on each participant’s T1 image (P1: F, 20 yr; P2: M, 21 yr; P3: M, 21 yr; P4: F, 22 yr; P5: F, 18 yr; P6: F, 24 yr). CTC = cerebello-thalamo-cortical tract (left = purple, right = lilac), FAT = frontal aslant tract (left = blue, right = light blue), P = participant, F/M = female/male, yr = years of age, L = left, R = right.

**Figure 3.**
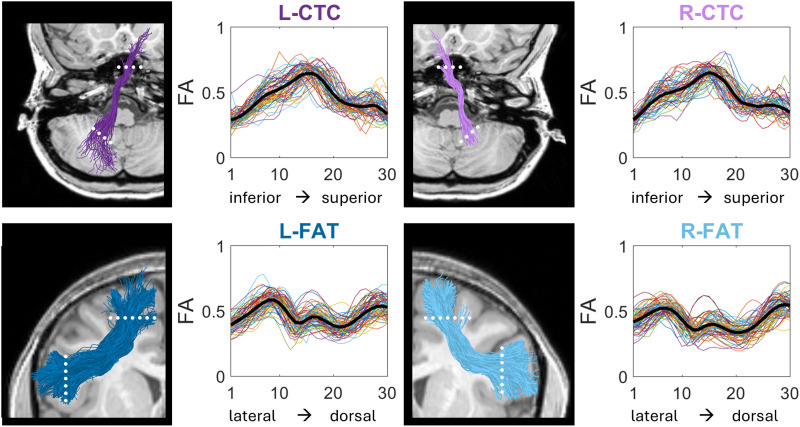
FA profiles along the tracts of interest. For each tract of interest, FA values are plotted along 30 equidistant nodes between two waypoint ROIs. Each colored line in the line-graphs represents an individual participant, and the thick black line indicates the mean FA-profile, averaged across participants. On the left of each plot, the relevant tract is visualized in a single participant (F, 18 yr), with dotted lines indicating the locations of the two ROIs. FA = fractional anisotropy, CTC = cerebello-thalamo-cortical tract, FAT = frontal aslant tract, L = left, R = right.

Tracts of interest were identified in the majority of participants (see Figure 2 for a visualization of the segmented tracts in six participants). Specifically, the left and right FAT were identified in the entire sample (*N* = 73), the left CTC was identified in 65 participants, and the right CTC in 54 participants. In participants for whom the CTC was not detected, visual inspection of the fibers in the native space suggested that the streamlines failed to cross to the contralateral hemisphere. Following tracts through a decussation is a well-known problem in tractography (He et al., 2021). Participants for whom a specific tract could not be identified were excluded from further analyses involving that tract. Visual inspection of all the identified tractograms in the native space of each individual confirmed that the tracts were segmented correctly, meeting anatomical expectations (Catani et al., 2012; Oishi et al., 2010) and consistent with a previous study that used the same ROIs and tractography methods in an independent sample (Jossinger et al., 2024). Indeed, the values and shapes of the FA profiles (see Figure 3) were also consistent with previous findings (Jossinger et al., 2024). No outlier tract-FA values were observed. Based on these quality assurance checks, all the identified tracts were included in the analyses.

### Spelling Associations With Tracts of Interest

**Figure 4.**
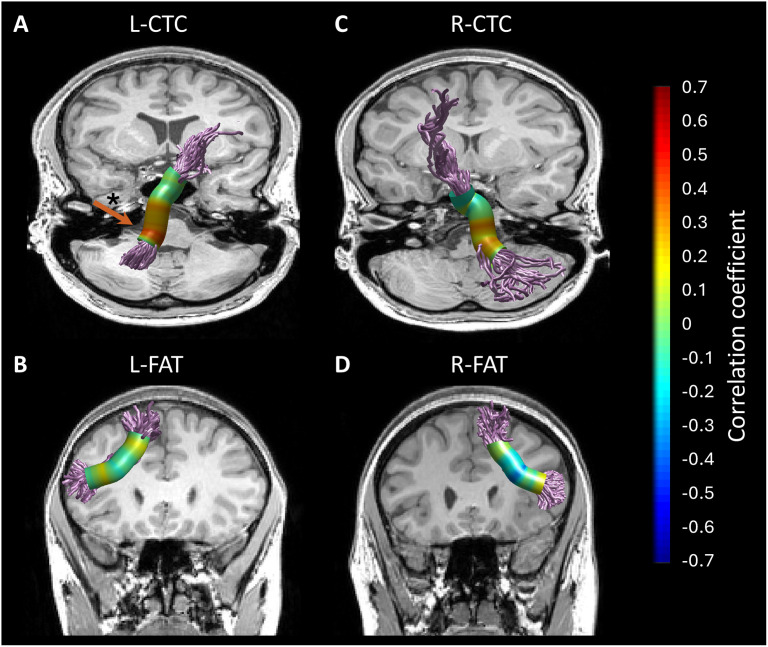
Correlations between spelling accuracy and FA along the tracts. Spearman’s correlation coefficients are visualized in 30 equidistant nodes along the tract cores. An arrow indicates the location of the cluster (nodes 3–9) that shows a significant correlation in the left CTC (*p* < 0.05, family-wise error corrected for 30 nodes). FA = fractional anisotropy, CTC = cerebello-thalamo-cortical tract, FAT = frontal aslant tract, L = left, R = right.

**Figure 5.**
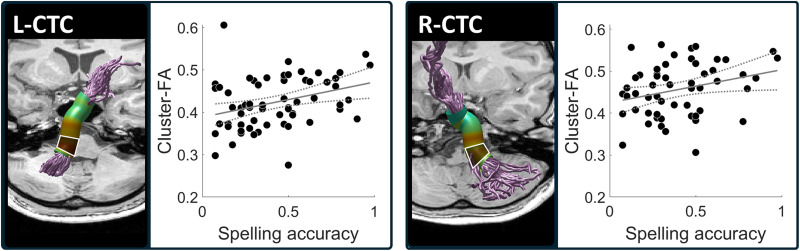
Correlations with spelling accuracy in the bilateral CTC tracts. Scatter plots depict the association between spelling accuracy and mean FA within the cluster of nodes showing a significant correlation in the left CTC (left scatter plot) and its homologous location in the right CTC (right scatter plot). Gray lines represent the best linear fit, enclosed by the 95% confidence interval (dashed lines). These scatter plots are for visualization purposes (significance and FWE correction were calculated along the tract; see Materials and Methods). The cluster locations are marked on the tractograms with framed shaded regions. CTC = cerebello-thalamo-cortical tract, FA = fractional anisotropy, L = left, R = right.

First, we evaluated the associations between spelling accuracy and Tract-FA (see Materials and Methods). This analysis did not yield significant correlations (see Table S1 for the full correlation matrix). Since FA values vary considerably along the trajectory of the tracts, but the shapes of the FA profiles are generally consistent across individuals, valuable insights may be gained by analyzing neurocognitive associations along the trajectory of the tract, rather than on the mean tract-FA values (Figure 3; Jossinger et al., 2024; Kruper et al., 2021; Palesi et al., 2016; Yeatman et al., 2012; see also Neurocognitive Association in a Localized Cluster Within the CTC in the Discussion section). We therefore performed a more sensitive node-by-node analysis, which evaluated the correlations between spelling and FA in each node along the tract, allowing for the detection of local associations in specific portions of the tracts. This analysis revealed that spelling accuracy was positively and significantly correlated with FA in a cluster of nodes in the left CTC (*p* < 0.05, FWE corrected for 30 nodes), such that better spellers had higher FA in that cluster of nodes (see Figure 4A). Positive correlations were also observed along the right CTC, but these clusters did not survive FWE correction. No significant correlations with spelling were detected along the bilateral FAT (see Figure 4B and D). The similar pattern of spelling-associations observed in the left and right CTCs is visualized in Figure 5, for the significant cluster detected in the left CTC, and for a homologous location in the right CTC.

### Lateralization and Specificity of Spelling Associations

To evaluate the lateralization of white matter associations with spelling, we constructed an LME model, with Hemisphere, Tract, and Spelling accuracy as predictors, and Tract-FA as the predicted variable (see Materials and Methods). A significant interaction between Spelling accuracy and Hemisphere would indicate lateralization. Instead, we found a significant interaction effect between Spelling accuracy and Tract (*p* < 0.001), but no significant interaction effect between Spelling accuracy and Hemisphere, nor between Spelling accuracy, Tract and Hemisphere (*p* > 0.4) (Table 2; see Table S2 for the full model). These results suggest that the correlations between spelling and Tract-FA are specific to the CTC, not FAT, but are not specific to the left hemisphere. Taken together, these results suggest bilateral involvement of the CTCs in spelling.

**Table 2.** Predictors for tract-FA estimated using a linear mixed-effects model with Participant as a random factor

Fixed effect	Estimate (*SE*)	*p*
(Intercept)	0.4541 (0.004)	<0.0001
Tract × Spelling accuracy	−0.0198 (0.006)	0.0005***
Hemisphere × Spelling accuracy	0.0042 (0.006)	0.4550
Tract × Hemisphere × Spelling accuracy	0.0019 (0.006)	0.7345

*Note*. See Table S2 for the full model. *SE* = standard error.

*** *p* < 0.001.

Next, we assessed the specificity of the associations detected in the CTCs to written versus spoken production. To this end, we evaluated multiple-regression models that predicted cluster-FA in the left or right CTC based on both Spelling accuracy and RAN scores. In both tracts, Spelling accuracy emerged as the primary significant predictor of cluster-FA. While RAN contributed uniquely to the models, it did so to a lesser extent compared to Spelling accuracy (Table 3). This interpretation was supported by nested model comparisons, which showed that adding Spelling accuracy to a RAN-only model significantly improved fit for the left CTC (*F*(1, 62) = 7.78, *p* = 0.007) and right CTC (*F*(1, 51) = 5.55, *p* = 0.022). Adding RAN to a Spelling-only model also significantly improved model fit for both tracts, but to a lesser extent (left CTC: *F*(1, 62) = 5.10, *p* = 0.028; right CTC: *F*(1, 51) = 4.49, *p* = 0.039). A comparison of the percentage of variance explained (Δ*R*^2^) further confirmed that Spelling accuracy accounted for more unique variance than RAN: Δ*R*^2^ = 10.3% for Spelling versus 6.8% for RAN in the left CTC, and Δ*R*^2^ = 9.2% for Spelling versus 7.5% for RAN in the right CTC. VIF values were close to 1, indicating no issues with multicollinearity (Johnston et al., 2018) (see also Figure S2 for the relationship between Spelling accuracy and RAN scores).

**Table 3.** Contribution of written and spoken language production measures to explaining FA in the CTC tracts

Predictor	B	*SE*	*β*	*t*	*p*	VIF
Left CTC						
(Constant)	0.435	0.025	–	17.410	<0.001	–
Spelling	0.081	0.029	0.322	2.789	0.007**	1.001
RAN	−0.005	0.002	−0.260	−2.245	0.028*	1.001
Overall regression: *R*^2^ = 0.18, *F*(2, 62) = 6.6, *p* = 0.002						
Right CTC						
(Constant)	0.475	0.029	–	16.519	<0.001	–
Spelling	0.085	0.036	0.305	2.356	0.022*	1.008
RAN	−0.006	0.003	−0.274	−2.129	0.039*	1.008
Overall regression: *R*^2^ = 0.15, *F*(2, 51) = 4.6, *p* = 0.014						

*Note*. Multiple regression models predicting cluster-FA in the left and right CTC from spelling accuracy scores and RAN scores. Cluster-FA refers to the mean FA across nodes 3–9 (see Figure 5). RAN scores are the average scaled scores of RAN Letters and RAN Digits. FA = fractional anisotropy, CTC = cerebello-thalamo-cortical tract, RAN = rapid automatized naming, B = unstandardized coefficient, *SE* = standard error, *β* = standardized coefficient, VIF = variance inflation factor.

* *p* < 0.05.

** *p* < 0.01.

Following this finding, we further examined the pairwise correlations between RAN and FA in the same tracts of interest. We found that RAN scores were not significantly correlated with FA in the bilateral CTCs, neither at the tract-FA level nor at the FA-profile level. In the left FAT, however, RAN scores were moderately correlated with tract-FA (*r* = −0.27, *p* = 0.022, FDR controlled at *q* < 0.1 for four tracts; Figure S3), consistent with previous research showing associations between the left FAT and speech production measures (Dick et al., 2019; see Table S3 for the full correlation matrix between RAN scores and tract-FA).

## DISCUSSION

This study evaluated the contributions of known speech-production pathways to written word production. Specifically, we assessed the associations between spelling performance and anisotropy values in the bilateral CTC and FAT in healthy university students. We found that spelling performance significantly correlated with anisotropy values in the left CTC. A similar trend was observed in the right CTC and no significant lateralization effects were found, suggesting a bilateral involvement of the CTC tracts in spelling. Additionally, associations of spelling accuracy with the CTCs remained significant when controlling for a spoken production measure (RAN). These findings extend the current understanding of the contribution of cerebello-cerebral pathways to language production across modalities, demonstrating the involvement of the CTC in central aspects of written word production. In contrast, no significant correlations were found between spelling performance and the bilateral FAT. Instead, the left FAT showed a moderate correlation with RAN scores, supporting the view that the left FAT is involved in speech fluency.

### Cerebello-Cerebral Contribution to Written Word Production

The present study showed a significant association between spelling accuracy scores and FA values in the left CTC. The CTC is the major output pathway of the cerebellum, transmitting information to contralateral cortical regions via the thalamus. Previous studies have demonstrated the involvement of the CTC in various speech production tasks, showing microstructural associations with overt reading, naming, and phonemic fluency (Bruckert et al., 2020; Jossinger et al., 2024; Keser et al., 2023; Travis et al., 2015). Our results extend these findings by demonstrating that information conveyed by the CTC also contributes to spelling performance in a written word production task. This aligns with consistent evidence from lesion and functional neuroimaging studies showing the involvement of the cerebellum in written word production (Planton et al., 2013; Purcell, Turkeltaub, et al., 2011; van Dun et al., 2016).

The specific processes carried out by the cerebellum in the context of written word production tasks are not well understood, partially due to the highly variable phenotypes of writing impairments observed in cases of cerebellar damage. The ongoing debate questions the extent to which the cerebellum plays a role in central, higher-level cognitive processes or in more peripheral graphomotor ones. Our study examined spelling accuracy in a difficult spelling-to-dictation task to assess central aspects of written word production. The stimuli consisted of low-frequency, irregularly spelled words, which require efficient lexical spelling processes, and the task was designed to minimize motor-related errors (e.g., the task was untimed and participants were allowed to correct their responses). Thus, the associations we detected between spelling accuracy and the CTC likely reflect the cerebellum’s contribution to central spelling processes rather than peripheral aspects (but see Limitations and Future Directions section, below). This interpretation aligns with a case study that reported disruptions to central spelling processes following cerebellar damage, specifically describing deficits in lexical spelling processes, characterized by phonologically plausible errors in irregular words (Mariën et al., 2009).

Other types of central writing impairments related to cerebellar damage include orthographic working memory deficits, evidenced by decreased letter accuracy on longer words and letter errors (e.g., letter additions, deletions, transpositions) resulting in phonologically implausible nonwords (Paul et al., 2022). Another cerebellar case study describes the non-linear spelling phenomenon where letters are not produced in the order they appear in the word (Lupo et al., 2019). In line with these reports, a possible account for the spelling association we detected with the CTC could be related to cerebellar involvement in sequencing. According to the sequence detection theory, the cerebellum is specialized in detecting and predicting repetitive patterns of temporally and spatially structured events and recognizes sequence violations (Braitenberg et al., 1997; Leggio & Molinari, 2015).

This hypothesis can be viewed within the cerebellar internal models theory, which posits that by interacting with the cerebral cortex, the cerebellum encodes and updates internal models for both motor and nonmotor tasks, including higher-level cognitive functions and language, to improve performance and coordination (Ito, 2008; Kawato, 1999; Shadmehr & Krakauer, 2008; Wolpert et al., 1998). In the context of language, the theory suggests that the cerebellum applies predictive and adaptive mechanisms to enhance fluency and accuracy in communication (Argyropoulos, 2016). Within this framework, the cerebellar circuitry may contribute to learning letter sequences that give rise to stable orthographic representations, necessary for successful spelling performance.

### Lateralization of the Spelling Associations With the CTC

The general picture that emerges from our analyses suggests that the spelling associations in the CTC are bilateral (see Table 2 and Figure 5). This finding is not in line with our hypotheses; we anticipated finding spelling associations primarily with the right CTC, based on prior fMRI and lesion studies predominantly linking the right cerebellar hemisphere and the left cerebral cortex with written language production (De Smet et al., 2011; Mariën et al., 2007, 2009; Paul et al., 2022; Planton et al., 2013; Purcell, Turkeltaub, et al., 2011; Silveri et al., 1999). However, other lesion studies document the involvement of the left cerebellar hemisphere in written word production (Fabbro et al., 2000, 2004; Lupo et al., 2019; Silveri et al., 1997). Together, these findings highlight the importance of both cerebellar hemispheres in this process. Moreover, the right-lateralized cerebellar results in fMRI may reflect input signals to the cerebellum rather than outputs from the cerebellum to the cortex, as suggested by neurophysiological studies of blood oxygen level-dependent (BOLD) signals in the cerebellum (Thomsen et al., 2004, 2009).

The left CTC transmits information to right cortical regions, including frontal and parietal areas (Behrens et al., 2003; Clower et al., 2001; Kelly & Strick, 2003; Middleton & Strick, 2001; Palesi et al., 2015). Therefore, the somewhat stronger correlation we observed in the left CTC (compared to the right) may reflect the importance of information flow to right-hemispheric regions involved in spelling. Prior functional and diffusion MRI studies have demonstrated the major role of the right cerebral hemisphere in spelling among both typical and atypical populations (Gebauer et al., 2012; Neophytou et al., 2023; Planton et al., 2013; Purcell, Turkeltaub, et al., 2011; Sagi et al., 2024). For example, Gebauer et al. (2012) found that children with spelling impairment had reduced FA in right hemispheric regions, which increased following spelling intervention. The importance of the right dorsal frontoparietal white matter pathway in spelling has also been implicated in studies of healthy adults (Sagi et al., 2024) and of children with reading and spelling deficits (Banfi et al., 2019; Gebauer et al., 2012). Because the correlation we observe with spelling is localized in the inferior segment of the CTC, we cannot determine the specific cortical regions receiving cerebellar inputs related to spelling. However, we hypothesize that inputs from the left cerebellar hemisphere via the CTC may modulate spelling-related activity in right frontal and parietal cortical regions and their interactions.

### Cerebellar Contributions to Written and Spoken Production

This study focused on tracts previously linked to speech production and assessed their associations with written word production. In addition to spelling accuracy, which probed written word production, we included RAN scores as an index of speeded spoken production to evaluate the potential contribution of speech production processes to the observed effects. Regression model comparisons indicated that spelling was the strongest predictor of FA in the CTCs; however, RAN also made a significant and unique contribution to explaining variance in FA. These findings suggest that the associations between CTC microstructure and spelling are not solely attributable to spoken production demands captured by RAN. Instead, RAN and spelling appear to account for distinct components of variability in CTC white matter properties.

To better understand these results, recall that the observed correlation is localized within a tightly packed segment of the CTC which later diverges into separate bundles. These different bundles may connect with distinct cortical projection zones, each involved in unique aspects of language production. Hence, different branches may carry signals relevant for different modalities of language production, spoken and written. Admittedly, studying long-range white matter bundles with distributed endpoints such as the CTC is unlikely to reveal exclusive one-to-one tract-to-function mapping. Future studies using more targeted tasks that manipulate specific aspects or stages within language production will be helpful in further understanding the level of cognitive specificity of the cerebello-cerebral connections.

### The Involvement of the FAT in Language Production

No significant correlations were identified in our data between spelling accuracy and FA in the bilateral FAT. Instead, the left FAT showed a moderate correlation with RAN scores. Although this effect was not strong enough to meet the standard FDR criterion (*q* < 0.05), it aligns with numerous studies that have demonstrated the involvement of the left FAT in speech fluency, both in clinical populations and in healthy individuals (Blecher et al., 2019; Bruckert et al., 2019; Catani et al., 2013; Jossinger et al., 2024; Li et al., 2017; Mandelli et al., 2014; Yablonski et al., 2021). Anatomically, the FAT connects the IFG and ventral precentral cortex with the SMA and pre-SMA, regions known for their involvement in both spoken and written language production (Alario et al., 2006; Planton et al., 2013; Purcell, Turkeltaub, et al., 2011). While the FAT may not be associated with the central aspects of written word production probed by our spelling task, it may be more relevant to peripheral aspects of written word production, such as typing rate or typing fluency. Indeed, the FAT has been implicated in the timing, planning, and coordination of voluntary motor sequences (Dick et al., 2019). Future studies should assess the role of the FAT in peripheral writing processes by collecting data related to kinematic aspects, such as timed writing or typing rate.

### Neurocognitive Association in a Localized Cluster Within the CTC

The significant FA-correlation with spelling detected in the left CTC was found in a cluster within the inferior segment of the tract. The correlation with mean tract-FA, however, did not reach statistical significance. It is not surprising that improved spatial resolution, provided by node-by-node analysis followed by correction for multiple comparisons, enhances sensitivity. Importantly, microstructural properties, like FA, are affected by several tissue factors, including axonal density, myelin, and directional coherence (Assaf & Pasternak, 2008), all of which may vary significantly along white matter tracts in a systematic manner (Kruper et al., 2021; Yeatman et al., 2012). Averaging over this variability may result in a loss of valuable information. When a significant cluster is found within a tract, it may indicate that this portion of the tract is homogeneous enough to reveal a significant correlation that exists throughout its length, while in the rest of the tract the association is masked by noise (e.g., crossing fibers, fanning streamlines or diverging bundles). Alternatively, it could be that this portion of the tract has undergone local changes in myelin, density, or axonal diameter, and these changes affect spelling performance, for example, by synchronizing signals arriving from distant regions (Fields, 2014; Osso & Hughes, 2024). These local changes may still affect the conductance properties of the whole tract. In sum, analyzing associations along the tract provides improved sensitivity, but the local associations detected in this way are likely to reflect the relevance of the tract as a whole to behavior.

### Limitations and Future Directions

Several limitations of this study should be acknowledged. First, the right CTC was identified in 75% of participants, compared to the 90% identification rate for the left CTC. Our segmentation protocol, using CSD-based probabilistic tractography, identifies the CTC as it decussates to the contralateral hemisphere, in accordance with the anatomy of this tract. In a prior study with an independent sample, this protocol successfully identified the CTC bilaterally in all participants (Jossinger et al., 2024). Possible reasons for the partial identification in the current dataset could be related to scanning parameters, such as the fact that slightly larger voxels were acquired in the current study. Alternative approaches for CTC segmentation were used in previous studies, including analyzing only its inferior segment below the decussation (e.g., Jossinger et al., 2021) or segmenting the inferior and superior segments separately and then combining them (e.g., Keser et al., 2023). To assess the reliability of our findings, we applied the first method to our data and segmented the inferior segment of the CTC below the decussation using tensor-based deterministic tractography. Using this strategy, the bilateral CTCs were identified in all participants, and the spelling associations remained consistent with those identified with the probabilistic tracts (see Figure S4). These findings are encouraging as they demonstrate the generalization of the key pattern of results across two different analysis pipelines.

Second, and relatedly, with the current segmentation tools we are able to reconstruct only the portion of the CTC tracts between the cerebellum and the contralateral thalamus, but not their cortical projections. During whole-brain tractography, streamlines extending beyond the white matter mask are truncated. Therefore, the reconstructed fibers are terminated upon entering the thalamic nuclei. Consequently, while our results indicate the involvement of the CTC in spelling processes, we cannot determine the specific cortical regions receiving cerebellar inputs related to spelling. Based on studies using virus retrograde transport techniques in primates (Clower et al., 2001; Kelly & Strick, 2003; Middleton & Strick, 2001; Stepniewska et al., 1994) and some tractography studies in humans (Behrens et al., 2003; Palesi et al., 2015), the CTC is expected to project to parietal and frontal regions, consistent with known spelling-associated areas. However, to better characterize the locations and nature of cerebellar contributions to spelling computations in the cortex, further studies employing functional connectivity analyses or targeted tractography between the thalamus and cortical regions will be required.

Third, in this study, we assessed spelling performance using a typed spelling-to-dictation task to address central spelling processes. Typed and handwritten productions have been shown to largely share central cognitive processes (Weingarten et al., 2004) and neural correlates (Higashiyama et al., 2015; Magrassi et al., 2010; Purcell, Napoliello, & Eden, 2011). We chose typing as it is the most commonly used modality for written production among adults nowadays (Pinet et al., 2022; Rapp, 2019). However, we acknowledge that participants’ responses might have included motor-related (peripheral) typing errors that cannot be reliably distinguished from spelling errors, potentially influencing accuracy scores.

Nonetheless, typing errors were less likely in the current task for several reasons. First, there was no time limit per trial, reducing the likelihood of speed-related typos. Second, typed responses were displayed on the screen, and participants were allowed to correct motor-related errors in real time. Furthermore, an analysis of spelling errors from this dataset (Sagi et al., 2024) indicated that errors were predominantly phonologically plausible (see Figure 1B for examples), which are unlikely to result from motor errors (Goodman & Caramazza, 1986). Nevertheless, future studies should aim to separate peripheral from central spelling measures more effectively, possibly by controlling for the baseline typing skills of participants.

Finally, the neurocognitive associations observed in the current study are based on English spelling, which involves a substantial degree of unpredictability in the correspondence between spoken and written forms. The extent to which these findings generalize to other orthographies, particularly those with more predictable sound-to-letter mappings, remains an open empirical question, to be addressed in future research.

### Conclusion

The associations detected in this study between anisotropy values in the cerebello-thalamo-cortical pathway and spelling performance in healthy adults suggest that cerebellar outputs to the cerebrum contribute to written word production. This finding aligns with the notion that connectivity between the cerebellum and the neocortex plays a role in higher-level language functions, demonstrating its importance to spelling processes for the first time. Additionally, our findings further support the established role of the cerebello-cerebral pathways in speech production but show that this association alone does not account for the spelling associations detected in the CTC. Furthermore, the current findings indicate that the frontal aslant tract may be more selectively involved in speech fluency rather than in central aspects of written word production. These results broaden our understanding of the white matter pathways involved in spelling, beyond the dorsal and ventral “classical” language-related pathways recently associated with spelling. Further research is needed to delineate the specific processes in spelling mediated by the cerebellar pathways and the potential involvement of the FAT in peripheral aspects of written word production.

## ACKNOWLEDGMENTS

This study was conducted as part of Romi Sagi’s doctoral dissertation, under the supervision of Professor Michal Ben-Shachar at the Gonda Multidisciplinary Brain Research Center, Bar-Ilan University. Brenda Rapp received funding from the Therapeutic Cognitive Neuroscience Division, Department of Neurology, Johns Hopkins Medical and from anonymous donors.

## FUNDING INFORMATION

Kathleen Rastle, Economic and Social Research Council (https://dx.doi.org/10.13039/501100000269), Award ID: ES/L002264/1. Michal Ben-Shachar, Israel Science Foundation (https://dx.doi.org/10.13039/501100003977), Award ID: 1083/17.

## AUTHOR CONTRIBUTIONS

**Romi Sagi**: Conceptualization: Lead; Formal analysis: Lead; Investigation: Lead; Methodology: Lead; Project administration: Lead; Validation: Lead; Visualization: Lead; Writing – original draft: Lead; Writing – review & editing: Lead. **Sivan Jossinger**: Conceptualization: Lead; Formal analysis: Supporting; Investigation: Supporting; Methodology: Equal; Software: Lead; Visualization: Supporting; Writing – review & editing: Equal. **J. S. H. Taylor**: Conceptualization: Supporting; Data curation: Lead; Investigation: Equal; Methodology: Equal; Writing – review & editing: Equal. **Kyriaki Neophytou**: Conceptualization: Supporting; Writing – review & editing: Equal. **Brenda Rapp**: Conceptualization: Equal; Funding acquisition: Supporting; Resources: Supporting; Writing – review & editing: Equal. **Kathleen Rastle**: Conceptualization: Equal; Data curation: Equal; Funding acquisition: Lead; Resources: Supporting; Writing – review & editing: Equal. **Michal Ben-Shachar**: Conceptualization: Lead; Funding acquisition: Supporting; Investigation: Supporting; Methodology: Supporting; Resources: Lead; Supervision: Lead; Validation: Supporting; Visualization: Supporting; Writing – original draft: Supporting; Writing – review & editing: Lead.

## DATA AND CODE AVAILABILITY STATEMENTS

The dMRI data were analyzed using open-source software, including mrDiffusion (https://github.com/vistalab/vistasoft), mrTrix3 (Tournier et al., 2019; https://www.mrtrix.org), and AFQ (Yeatman et al., 2012; https://github.com/yeatmanlab/AFQ). Code and ROIs for the segmentation of the CTC and FAT can be found at https://github.com/yeatmanlab/AFQ/tree/master/cp-prob_segmentation and https://github.com/yeatmanlab/AFQ/tree/master/aslant, respectively. Behavioral and imaging data are available at https://osf.io/mv2nd/?view_only=b6b9d0a4b52b4fc1adb294f0091c2915.

## Supplementary Material



## TECHNICAL TERMS

Central spelling processes: Cognitive processes involved in mapping phonological word forms and/or meanings to orthographic word forms, such as retrieving lexical orthographic representations from long-term memory, converting phonemes (speech sounds) into graphemes (letters), and holding these in working memory prior to production. These processes are independent of the output modality or format (e.g., typing, handwriting, oral spelling).
Peripheral spelling processes: Processes involved in programming and executing motor commands required to produce spellings, specific to the output modality (e.g., handwriting, typing).
Diffusion MRI (dMRI): An MRI-based imaging method that measures the speed and directionality of water diffusion in biological tissues.
Constrained spherical deconvolution (CSD): A computational model of dMRI data that estimates the distribution of fiber orientations within each voxel.
Tensor modeling: A computational model describing the speed and directionality of water diffusion, represented as an ellipsoid fit to dMRI data in each voxel.
Fractional anisotropy (FA): A measure of the degree of directional diffusion, based on the tensor model.
Tractogram: A 3D representation of virtual fibers generated from dMRI data.
Phonologically plausible error: A spelling response that corresponds to the sounds of a word but is orthographically incorrect (e.g., spelling *cat* as *KAT* or *yacht* as *YOT*).
