# Supplementary material for: Cerebello-Cerebral Pathways Contribute to Written Word Production
Source: Neurobiol Lang (Camb). 2025 Aug 14;6:nol.a.10. doi: 10.1162/nol.a.10 (PMC12373453; doi:10.1162/nol.a.10)
Supplement: Supplementary file 1 [file nol-6-1-10-s001.pdf]

Cerebello-cerebral pathways contribute to written word production

Supplementary Information

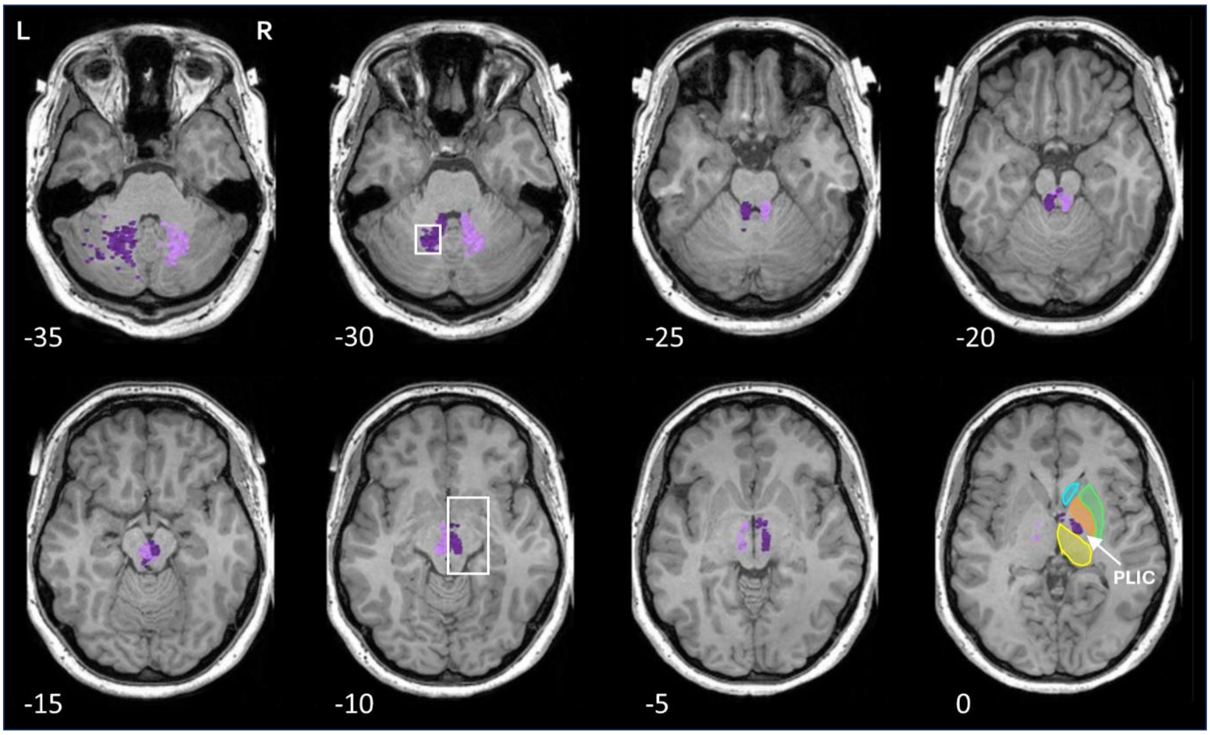

Figure S1. Anatomical trajectory of the bilateral CTC tracts. Trajectories of the left CTC (purple) and right CTC (lilac) are marked on T1 axial images of a representative participant (F, 20y). The CTCs emerge from each cerebellar hemisphere and project to the contralateral cerebral cortex through the contralateral thalamus (ventral lateral nucleus). They reach the contralateral thalamus after decussating at the level of the inferior colliculus and travel along the posterior limb of internal capsule (PLIC). Z coordinates are reported for each slice in MNI space (mm). Waypoint ROIs used to segment the CTC are denoted by white rectangles, presented for the L-CTC. Colored structures: R-thalamus (yellow), R-putamen (green), R-caudate nucleus (cyan), R-globus pallidus (orange). CTC – cerebello-thalamo-cortical tract, L – left, R – right.

Table S1

Spearman’s correlations between Spelling accuracy and tract-FA

Tract	r	*p*	*N*
Left CTC	0.23	0.0598	65
Right CTC	0.21	0.1212	54
Left FAT	0.03	0.7909	73
Right FAT	-0.13	0.2590	73

CTC – cerebello-thalamo-cortical tract, FAT – frontal aslant tract

Table S2
Predictors for tract-FA estimated using a linear mixed-effects model with Participant as a random factor.

Fixed effect	Estimate	SE	*t*	DF	*p*
(Intercept)	0.4541	0.0042	106.9	257	<0.0001
Tract	$5.5*10^{-6}$	0.0026	0.002	257	0.9983
Hemisphere	0.0053	0.0026	2.057	257	0.0406*
Spelling accuracy	0.0139	0.0129	1.423	257	0.1559
Tract*Hemisphere	0.0096	0.0096	3.706	257	0.0003***
Tract*Spelling accuracy	-0.0198	-0.0198	-3.544	257	0.0005***
Hemisphere*Spelling accuracy	0.0042	0.0041	0.748	257	0.4550
Tract*Hemisphere*Spelling accuracy	0.0019	0.0019	0.339	257	0.7345

SE – standard error, DF – degrees of freedom
* $p < 0.05$, *** $p < 0.001$

Figure S2. Relationship between written and spoken production measures. The scatter plot shows that Spelling accuracy and RAN scores are not correlated. The r value is Spearman's correlation coefficient.

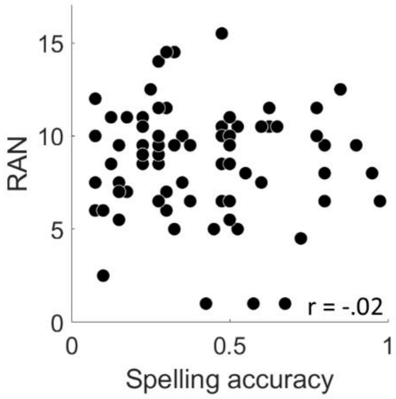

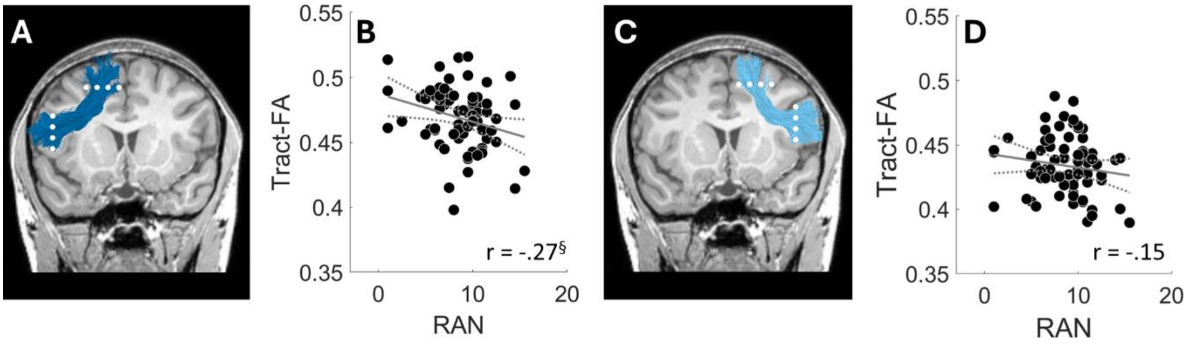

Figure S3. FAT associations with spoken language production. Tractograms demonstrate the left FAT (A) and right FAT (C) in a single participant (F, 18y) with dotted lines indicating the locations of the two ROIs. Tract-FA is calculated as the mean FA of 30 equidistant nodes between the two ROIs (see Methods). Scatter plots depict the association between RAN and tract-FA in the left (C) and right (D) FAT. Gray lines represent the best linear fit, enclosed by the 95% confidence interval (dashed lines). RAN scores are mean scaled-scores on the letter and digit subtests. The r values are Pearson's correlation coefficients. § $p < 0.05$, FDR controlled at 0.1 across four tracts. FAT – frontal aslant tract, FA – fractional anisotropy, RAN – rapid automatized naming.

Table S3

Pearson's correlations between RAN scores and tract-FA

Tract	r	*p*	*N*
Left CTC	-0.18	0.1589	65
Right CTC	-0.19	0.1659	54
Left FAT	-0.27	0.0216	73
Right FAT	-0.15	0.2126	73

CTC – cerebello-thalamo-cortical tract, FAT – frontal aslant tract

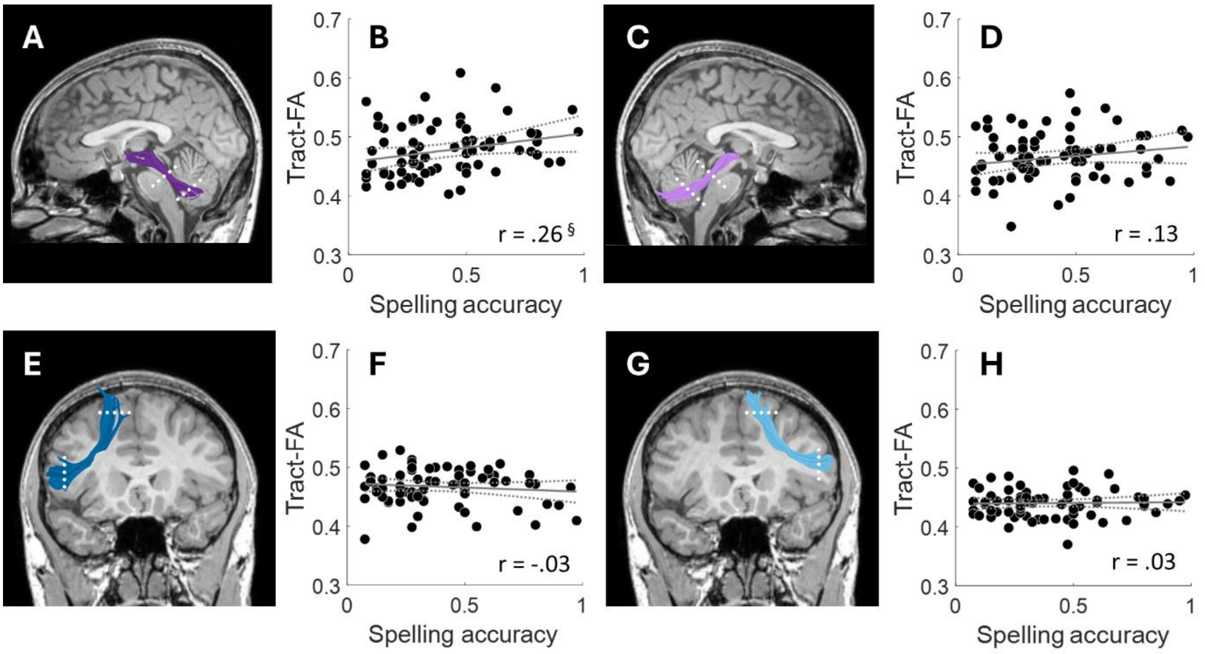

Figure S4. Spelling associations in tensor-based deterministic tractography. The inferior segment of the CTC (below the decussation) was identified using ROIs and tracking parameters as in Jossinger et al. (2019). This method allowed the identification of the bilateral CTC in the full sample (N = 73). The bilateral FAT were also identified in the full sample using deterministic tractography, but one outlier was removed for the left FAT due to an extreme tract-FA value (see Kronfeld-Duenias et al. (2016) for deterministic tracking parameters of the FAT). Tractograms demonstrate the left CTC (A), right CTC (C), left FAT (E) and right FAT (G) in an example participant (M, 19y), with dotted lines indicating the locations of the two ROIs between which FA was averaged. Scatter plots depict the association between spelling accuracy and tract-FA in the left CTC (B), right CTC (D), left FAT (F) and right FAT (H). The association patterns remained similar to the CSD-based probabilistic tracking results. Gray lines represent the best linear fit, enclosed by the 95% confidence interval (dashed lines). The r values are Spearman's correlation coefficients. § $p < 0.05$, FDR controlled at 0.1. FA – fractional anisotropy, CTC – cerebello-thalamo-cortical, FAT – frontal aslant tract.
